# Neuropsychological symptoms in workers handling cargo from shipping containers and export logs

**DOI:** 10.1007/s00420-022-01870-8

**Published:** 2022-05-06

**Authors:** Ruth Hinz, Andrea ’t Mannetje, Bill Glass, Dave McLean, Jeroen Douwes

**Affiliations:** Research Centre for Hauora and Health, Massey University, Wellington, New Zealand

**Keywords:** Neuropsychological symptoms, Shipping containers, Fumigation/fumigant, Volatile organic compounds, Occupational groups, Workplace exposure

## Abstract

**Purpose:**

Acute poisonings of workers handling shipping containers by fumigants and other harmful chemicals off-gassed from cargo have been reported but (sub)-chronic neuropsychological effects have not been well studied.

**Methods:**

This cross-sectional study assessed, using standardised questionnaires, current (past 3-months) neuropsychological symptoms in 274 container handlers, 38 retail workers, 35 fumigators, and 18 log workers, all potentially exposed to fumigants and off-gassed chemicals, and a reference group of 206 construction workers. Prevalence odds ratios (OR), adjusted for age, ethnicity, smoking, alcohol consumption, education, personality traits and BMI, were calculated to assess associations with the total number of symptoms (≥ 3, ≥ 5 or ≥ 10) and specific symptom domains (neurological, psychosomatic, mood, memory/concentration, fatigue, and sleep).

**Results:**

Compared to the reference group, exposed workers were more likely to report ≥ 10 symptoms, statistically significant only for retail workers (OR 6.8, 95% CI 1.9–24.3) who also reported more fatigue (OR 10.7, 95% CI 2.7–42.7). Container handlers with the highest exposure-duration were more likely to report ≥ 10 symptoms, both when compared with reference workers (OR 4.0, 95% CI 1.4–11.7) and with container handlers with shorter exposure duration (OR 7.5, 95% CI 1.7–32.8). The duration of container handling was particularly associated with symptoms in the memory/concentration domain, again both when compared to reference workers (OR 8.8, 95% CI 2.5–31.4) and workers with the lowest exposure-duration (OR 6.8, 95% CI 1.5–30.3).

**Conclusion:**

Container handlers may have an increased risk of neuropsychological symptoms, especially in the memory/concentration domain. Retail workers may also be at risk, but this requires confirmation in a larger study.

## Introduction

The design of sealed shipping containers allows for only limited natural ventilation during transport (Svedberg and Johanson 2017). As a result, fumigants (e.g. ethylene oxide, methyl bromide, phosphine, chloropicrin), used for biosecurity reasons or to prevent damage to cargo, and chemicals off-gassed from cargo or packaging (e.g. formaldehyde, toluene, benzene) may accumulate in the air, potentially reaching unsafe levels for workers unloading or inspecting these containers (European Agency for Safety and Health at Work 2018). Indeed, high levels of these chemicals have been found in the air of sealed containers and acute poisonings in container handlers have been reported (Baur 2015; Breeman 2009; Budnik et al. 2012; European Agency for Safety and Health at Work 2018; Kloth et al. 2014; Preisser et al. 2012, 2011; Roberts et al. 2014; Spijkerboer et al. 2008; Verschoor et al. 2010, 2011), ranging from headaches to coma. In addition to container handlers, inspectors and fumigators, retail workers, bystanders and consumers may also be exposed potentially resulting in health effects also in those groups (Baur et al. 2010; Budnik et al. 2017; Knol-de Vos et al. 2005; Preisser et al. 2012; Preisser et al. 2011).

While acute neurotoxicity has been the primary concern, it is possible that chronic health effects, associated with long-term exposure to these chemicals, such as sustained memory and concentration deficits, fatigue, and severe persistent neurological outcomes, may also occur. In particular, many of these chemicals (e.g. methyl bromide, 1,2-dichloroethane) are known, or suspected, to cause both acute and chronic neuropsychological symptoms (European Agency for Safety and Health at Work 2018). However, research on long-term health effects in container handlers, fumigators, and retail workers is scant. One health survey among 125 French dockworkers and those working in related occupations reported an overall low prevalence of neuropsychological symptoms, which the authors suggested could be due to a healthy worker effect (Lucas et al. 2019). They also reported a positive association between fumigant exposure in the past years and memory disorders, but this was based on a very small sample, and analyses were not controlled for potential confounders. To our knowledge, no comprehensive epidemiological studies have been conducted in this workforce.

Globally, shipping container throughput has risen from 622 to 802 million twenty-foot equivalent units (TEU) between 2012 and 2019 (Statista 2020). In New Zealand, an island nation reliant on import and export via sea cargo ships, this sector employs 116,100 workers (Stats New Zealand 2020). Considering the volume of world trade and the size of the workforce, studies on the health risks of chemical exposures in workers employed in the container industry and those who fumigate and/or handle container goods are warranted.

This study assessed neuropsychological symptoms through standardised questionnaires in 274 container handlers and three other groups with potential exposure to fumigants and residual chemicals: 38 retail workers, 35 fumigators, and 18 log workers, who load export logs onto ships, some of which are fumigated. Symptom prevalence was compared with a reference group of 206 construction workers who are unlikely to be exposed to these chemicals. Internal comparisons within exposed workers to assess associations with duration of employment in these jobs were also conducted.

## Methods

### Study design

This study is a cross-sectional health survey that assessed neuropsychological symptoms in workers who handle shipping containers and export logs, retail workers, and fumigators, as well as a reference group of construction workers who are unlikely to be exposed to fumigants and chemicals off-gassed from container cargo. For this paper, container and export log workers, fumigators, and retail workers are described collectively as “exposed workers”.

Written consent was obtained from all participants and ethics approval was granted by the Multi-region Ethics Committee of the New Zealand Ministry of Health (MEC/12/02/010).

### Participant recruitment

Companies accredited to open and inspect (for biosecurity reasons) overseas containers were randomly selected from a list of Accredited Transitional Facilities (ATF), published by the New Zealand Ministry of Primary Industries. The companies that participated had a workforce size and total throughput of cargo approximately reflective of this industry in New Zealand., They comprised distribution centres (*n* = 8), third-party logistics providers (*n* = 22), and companies unloading their own imported containers (*n* = 48). We also recruited a government department involved in inspecting overseas containers (*n* = 1), a company specialising in export log operations (*n* = 1), port companies (*n* = 4), fumigation companies (*n* = 5) and a retail chain (*n* = 1). In total, 90 companies agreed to participate and 111 declined. Participants who inspected containers were combined with the container handler group as their tasks frequently overlapped.

The companies, situated at eleven locations throughout New Zealand, ranged in size from owner-operated to large distribution centres, with the number of participants mostly reflecting the size of the company (1–2 participants, 50 companies; 3–6 participants, 27 companies; and 7–110 participants, 13 companies). Management identified potential participants based on availability and workplace requirements resulting in 493 workers (321 container handlers, 110 retail workers, 21 log workers and 35 fumigators) participating in the study. In total, 38 workers declined to participate in the study of which 21 (56.8%) were woman (who were excluded from the analyses; see below). The response rate, based on all males invited to the study, was 96%.

A reference group (*n* = 222; response rate, 64%), which was not involved in container handling and/or fumigation, consisting of construction workers from various trades (scaffolders, carpenters, electricians, builders and building labourers, fire safety system installers, plumbers and associated management staff), was recruited throughout the North Island of New Zealand, with a focus on the main centres (Wellington and Auckland). Companies were identified from the Yellow Pages and Internet searches.

### Questionnaire

Current (i.e. in the past three months) neuropsychological symptoms were measured using an adapted version of the EUROQUEST questionnaire, which was developed to evaluate neuropsychological health effects associated with occupational exposure to neurotoxic chemicals (Karlson et al. 2000), including long-term solvent exposure (Carter 2002). EUROQUEST is widely used both in epidemiological studies and as a screening tool in clinical settings (Kaukiainen et al. 2008, 2009) and is well-validated against clinical criteria. In particular, evaluation studies have shown a factor structure consistent with the original intention, and reasonable to good internal consistency of domains and symptoms; it was also shown to be sensitive for exposures to neurotoxic chemicals, especially in the memory and concentration domain, as assessed by comparing results with neuropsychological tests(Carter 2002; Karlson et al. 2000; Kaukiainen et al. 2008, 2009; Rouch et al. 2003; Williamson 2007).

The questionnaire, which was administered face-to-face, consists of 59 questions covering the following symptom domains: neurological symptoms, psychosomatic symptoms, mood, memory/concentration, fatigue and sleep quality (see tables). In addition, six questions on acute symptoms that occurred in the last three months were included (see Tables). Symptom frequency for these and the 59 core symptoms was reported on a 4-point scale: ‘seldom or never’, ‘sometimes’, ‘often’ or ‘very often’. The EUROQUEST also contains eight questions on sensitivity to environmental conditions (see Tables) and six anxiety-related questions (‘I am generally a nervous person’, ‘I think I am generally less capable than others in overcoming my difficulties’, ‘I worry a lot about trivial things’, ‘I often feel that something bad might happen at any moment’, ‘I often feel that even trivial problems are too much for me’, ‘I usually feel insecure’) rated on a different 4-point scale: ‘strongly disagree’, ‘disagree’, ‘agree’ or ‘strongly agree’. The EUROQUEST assesses perceived general health in four questions (‘How good is your health’, ‘How is your health now compared to what it was 5 years ago’, ‘How do you feel about your life in general’, ‘How do you feel about your life now compared to five years ago’) rated on a 4-point scale: ‘very good’ ‘good’, ‘poor’ or ‘very poor’.

Symptoms were initially dichotomised with ‘strongly disagree’ or ‘disagree’, ‘seldom or never’ or ‘sometimes’, and ‘very good’ and ‘good’ comprising a negative response and ‘agree’ or ‘strongly agree’, ‘often’ or ‘very often’, and ‘poor’ or ‘very poor’ comprising a positive response (Kaukiainen et al. 2009). If this classification resulted in a positive response of < 5% (as was the case for 15 questions), resulting in insufficient power for subsequent analyses, a positive response was reclassified with ‘sometimes’, ‘often’ and ‘very often’ constituting a positive response. Likewise, if the initial classification resulted in a positive response of > 10% (seven questions), suggesting that the outcome response was insufficiently discriminatory, a positive response was reclassified with only ‘very often’ constituting a positive response.

Anxiety and perceived general health questions were used to calculate an aggregated individual personality trait score, which has been found to be associated with participants under or over reporting symptoms (Kaukiainen et al. 2009). The score was calculated by adding the scores of the six anxiety and four perceived general health questions, resulting in a potential range from 0 to 10, as we have done in previous studies (Keer et al. 2016). Analyses were controlled for this (see below). Additional questions were asked on demographics, work characteristics and potential confounders.

### Statistical analyses

All statistical analyses were conducted using Stata version 15.1 (StataCorp LP, Texas, USA). Because the reference group had only two female participants, and, apart from the retail workers, relatively few females (*n* = 33) were employed in the other occupational groups, we excluded all females (*n* = 107) from the analyses. Additionally, four container handlers were excluded from the exposed group because of additional exposure to welding fumes; 19 workers involved in container handling were excluded as they did not unload containers; and 13 reference workers were excluded because of exposure to paint fumes, and one reference worker did not complete the questionnaire. For the analyses, we therefore had data for 274 container handlers, 38 retail workers, 18 log workers, 35 fumigators and 206 reference workers.

Neuropsychological symptoms were analysed using two approaches: (1) grouped based on the total number of positive symptoms reported (≥ 3 symptoms, ≥ 5 symptoms, ≥ 10 symptoms); and (2) grouped based on the number of positive symptoms (≥ 3) in each symptom domain (described above), an approach previously shown to be highly sensitive and specific in the classification of chronic solvent neurotoxicity patients (Kaukiainen et al. 2009). We also considered non-aggregated individual neuropsychological symptoms (yes/no), as well as acute symptoms and sensitivity to environmental conditions. Prevalence odds ratios (OR), comparing symptoms between the exposed and the reference group, and between sub-groups of exposed workers and the reference group, were calculated using logistic regression.

The effect of exposure duration was assessed only for container handlers because the number of participants in the other occupations was too small. We used two approaches: (1) based on the years spent in the industry unloading containers; and (2) a more refined version of the first approach, based on the years spent in the industry combined with the actual time unloading containers per day, week or month. For approach 2, we calculated annual hours spent unloading containers and divided this by 1920, the total annual workable hours (48 weeks × 40 h/week) and multiplied this by the number of years employed in this industry, resulting in an “exposure years equivalent”. Both duration metrics were subsequently categorised in three exposure groups (low, medium, high) based on tertiles. Prevalence ORs were calculated comparing the symptom prevalence of container handlers in the three exposure duration categories with that in the reference group (external comparison). We also made internal comparisons (within the group of container handlers) by calculating prevalence ORs that involved comparing the two highest with the lowest exposure duration category.

Regression analyses were adjusted for age (in years), ethnicity (Māori and Pacific vs “other”, mostly New Zealand European), smoking (non-smoker, ex-smoker, current smoker), alcohol consumption (standard drinks per week), education status (primary/secondary education vs tertiary education/trade certificate), BMI (kg/m^2^) and personality trait score (based on questions about anxiety and general health; see above). For analyses involving individual symptoms, which often had a relatively low prevalence, ORs were adjusted for age and personality trait score only to avoid non-convergence.

## Results

Compared to the reference group, exposed workers consumed more alcohol and tobacco, received less education, were slightly older, scored higher in the personality traits scale, had a higher BMI, and were more likely to be Māori or of Pacific descent (Table 1). All analyses were controlled for these factors (with exception of analyses focused on individual symptoms; see above).

**Table 1:** Demographic and work characteristics of the exposed groups and reference group

	reference group (*n* = 206)		exposed group (*n* = 365)		exposed group by occupation							
					container handler (*n* = 274)		retail worker (*n* = 38)		log worker (*n* = 18)		Fumigator (*n* = 35)	
	*n*	%	*n*	%	*n*	%	*n*	%	*n*	%	*n*	%
Ethnicity												
Māori/Pacific	92	44.7	141	38.6	106	38.7	12	31.6	9	50.0	14	40.0
Other (incl. New Zealand European)	113	54.9	223	61.1	167	61.0	26	68.4	9	50.0	21	60.0
Smoking status												
Non-smoker	91	44.2	170	46.6	125	45.6	27	71.1	4	22.2	14	40.0
Ex-smoker	43	20.9	80	21.9	64	23.4	2	5.3	4	22.2	10	28.6
Current smoker	72	35.0	113	30.9	84	30.7	8	21.1	10	55.6	11	31.4
Education level												
Primary/secondary	104	50.5	239	65.5	184	67.2	17	44.7	16	88.9	22	62.9
Tertiary/trade cert	102	49.5	125	34.3	89	32.5	21	55.3	2	11.1	13	37.1

	Median	Range	Median	Range	Median	Range	Median	Range	Median	Range	Median	Range
Age	36.8	17.1–66.5	39.2	17.2–76.7	39.3	18.0–76.0	28.4	18.8–63.2	42.3	21.0–67.4	38.4	17.2–65.9
Alcohol (standard drinks/week)	6.0	0–80.0	12.2	0–140.0	8.0	0–85.0	6	0.0–52.0	14.5	1–140.0	8.8	0–44.0
EUROQUEST personality score	0	0–8	1	0–7	1	0–7	1	0.0–6	1	0–4	0	0–7
BMI	27.6	14.6–42.2	29.4	16.4–64.3	29.1	16.4–64.3	25.8	18.2–44.3	29.4	17.3–36.8	28.4	21.5–45.4
Working hours (hours/week)	40.0	3.0–100.0	40.0	7.0–80.0	40.0	7.0–70.0	40.0	9.0–60.0	45.0	20.0–80.0	40.0	10.0–60.0

The likelihood of reporting ≥ 3 symptoms and ≥ 5 symptoms overall was similar for exposed and reference workers (Table 2); however, exposed workers were more likely to report ≥ 10 symptoms, although this did not reach statistical significance (OR 2.0, 95% CI 0.9–4.7). Elevated ORs for ≥ 10 symptoms were observed for all exposed subgroups, reaching statistical significance for retail workers only (OR 6.8, 95% CI 1.9–24.3; Table 2). Exposed workers were also more likely to report ≥ 3 symptoms for the following two symptom domains: memory/concentration (OR 2.6, 95% CI 0.9–7.5) and fatigue (OR 2.3, 95% CI 0.9–6.2; Table 2). Increased risks for these domains were observed for all exposed sub-groups, but this was statistically significant only for fatigue in retail workers (OR 10.7, 95% CI 2.7–42.7; Table 2).

**Table 2:** Prevalence odds ratios (OR) of neuropsychological symptoms and symptom domains comparing exposed with reference workers

	Reference group (*n* = 206)	Exposed group (*n* = 365)		Exposed group by occupation (compared to the reference group)							
				Container handler (*n* = 274)		Retail worker (*n* = 38)		Log worker (*n* = 18)		Fumigator (*n* = 35)	
	*n* (%)	*n* (%)	OR (95% CI)	*n* (%)	OR (95% CI)	*n* (%)	OR (95% CI)	*n* (%)	OR (95% CI)	*n* (%)	OR (95% CI)
Number of symptoms											
≥ 3 symptoms	78 (37.9)	150 (38.9)	0.9 (0.6–1.3)	103 (36.9)	0.8 (0.5–1.2)	15 (39.5)	1.3 (0.6–2.9)	12 (66.7)	2.5 (0.8–7.4)	12 (34.3)	0.7 (0.3–1.7)
≥ 5 symptoms	45 (21.8)	98 (25.8)	1.1 (0.7–1.8)	69 (24.7)	1.0 (0.6–1.7)	10 (26.3)	1.5 (0.6–3.8)	8 (44.4)	2.4 (0.8–7.4)	7 (20.0)	0.9 (0.3–2.5)
≥ 10 symptoms	12 (5.8)	35 (9.6)	2.0 (0.9–4.7)	22 (7.9)	1.5 (0.6–3.7)	7 (18.4)	6.8 (1.9–24.3)**	2 (11.1)	2.0 (0.3–13.6)	4 (11.4)	2.6 (0.6–11.8)
Symptom domains (≥ 3)											
Neurological symptoms	9 (4.4)	20 (5.5)	1.1 (0.4–2.6)	13 (4.8)	0.9 (0.4–2.4)	3 (7.9)	2.0 (0.5–8.6)	3 (16.7)	3.0 (0.6–15.2)	1 (2.9)	–
Psychosomatic symptoms	20 (9.9)	39 (10.7)	0.8 (0.4–1.4)	29 (10.7)	0.7 (0.4–1.4)	5 (13.2)	1.3 (0.4–3.9)	1 (5.6)	0.3 (0.0–2.7)	4 (11.4)	0.5 (0.1–2.5)
Mood	11 (5.3)	29 (8.0)	1.2 (0.5–2.7)	21 (7.7)	1.1 (0.4–2.5)	4 (10.5)	2.7 (0.7–10.4)	1 (5.6)	0.8 (0.1–7.7)	3 (8.6)	1.3 (0.2–6.8)
Memory/concentration	8 (3.9)	27 (7.4)	2.6 (0.9–7.5)	18 (6.6)	2.2 (0.7–6.7)	4 (10.5)	4.4 (0.9–21.4)	2 (11.1)	3.2 (0.4–27.0)	3 (8.6)	3.3 (0.6–20.3)
Fatigue	8 (3.9)	27 (7.4)	2.3 (0.9–6.2)	17 (6.2)	1.6 (0.5–4.6)	7 (18.4)	10.7 (2.7–42.7)***	1 (5.6)	1.6 (0.1–18.1)	2 (5.7)	3.3 (0.6–19.8)
Sleep	5 (2.4)	18.(4.9)	1.5 (0.5–5.0)	14 (5.1)	1.6 (0.5–5.4)	1 (2.6)	1.2 (0.1–12.7)	1 (5.6)	1.4 (0.1–16.9)	2 (5.7)	1.4 (0.1–13.8)

All analyses were adjusted for age, ethnicity, smoking, alcohol consumption, education, personality traits and BMI

*p < 0.05, **p < 0.01, ***p < 0.001

In container handlers, we assessed whether the risk of symptoms increased by: (i) the number of years worked with containers; and (ii) the number of equivalent years unloading containers (Table 3). When comparing workers in the highest exposure tertiles of these exposure metrics with the reference group, the risk of reporting ≥ 10 symptoms overall increased, with ORs of 3.8 (95% CI 1.1–12.4) and 4.0 (95% CI 1.4–11.7), respectively. When comparing container handlers in the highest exposure tertile with those in the lowest, associations were more pronounced, with ORs of 91 (95% CI 4–2000) and 7.5 (95% CI 1.7–32.8) for each exposure metric, respectively. For the domains of psychosomatic symptoms, memory/concentration, and fatigue, odds ratios increased with duration using either exposure metric. However, most consistent trends were observed for the memory/concentration domain. In particular, using “number of years working with containers”, we found ORs of 10.7 (95% CI 2.4–46.9) and 174 (95% CI 7.5–4064) for the highest tertile when compared to the external reference group and lowest exposure tertile, respectively; using “equivalent years unloading”, ORs of 8.8 (95% CI 2.5–31.4) and 6.8 (95% CI 1.5–30.3) were found (Table 3).

**Table 3:** Prevalence odds ratios of neuropsychological symptoms and symptom domains for container handlers (*n* = 274) stratified by employment and exposure duration

	External comparison (compared to the reference group)							Comparison within container handlers (compared with lowest exposure duration)	
	*n* (%)	*n* (%)	OR (95% CI)	*n* (%)	OR (95% CI)	*n* (%)	OR (95% CI)	OR (95% CI)	OR (95% CI)
Number of years working with containers	reference (*n* = 206)	0.01–2.5 years (*n* = 92)		2.51–9.75 years (*n* = 91)		9.76–44.42 years (*n* = 91)		2.51–9.75 years (*n* = 91)	9.76–44.42 years (*n* = 91)
Number of symptoms									
≥ 3 symptoms	78 (37.9)	30 (10.9)	0.7 (0.4–1.2)	39 (14.2)	1.1 (0.6–1.9)	34 (12.4)	0.8 (0.4–1.5)	1.4 (0.7–2.9)	1.0 (0.5–2.3)
≥ 5 symptoms	45 (21.8)	14 (5.1)	0.4 (0.2–0.9)*	30 (10.9)	1.6 (0.8–2.9)	25 (9.1)	1.6 (0.8–3.3)	4.2 (1.7–10.8)**	5.1 (1.7–15.4)**
≥ 10 symptoms	12 (5.8)	1 (0.4)	0.1 (0.0–1.3)	11 (4.0)	2.0 (0.7–6.0)	10 (3.6)	3.8 (1.1–12.4)*	32.6 (2.0–543)*	91.4 (4.1–2,038)**
Symptoms domains (≥ 3)									
Neurological symptoms	9 (4.4)	5 (1.8)	1.4 (0.4–4.5)	2 (0.7)	0.5 (0.1–2.4)	6 (2.2)	1.3 (0.4–4.6)	0.4 (0.1–2.7)	1.1 (0.2–6.0)
Psychosomatic symptoms	20 (9.9)	6 (2.2)	0.5 (0.2–1.5)	9 (3.3)	0.6 (0.2–1.5)	14 (5.1)	1.2 (0.5–2.9)	0.9 (0.3–3.2)	2.1 (0.6–7.8)
Mood	11 (5.3)	3 (1.1)	0.3 (0.1–1.3)	11 (4.0)	1.8 (0.6–5.1)	7 (2.6)	1.6 (0.5–5.3)	9.4 (1.4–62.4)*	9.1 (1.1–77.7)*
Memory/concentration	8 (3.9)	1 (0.4)	0.1 (0.0–1.5)	8 (2.9)	3.0 (0.8–11.2)	9 (3.3)	10.7 (2.4–46.9)**	35.9 (2.3–565)*	174.4 (7.5–4,064)**
Fatigue	8 (3.9)	2 (0.7)	0.4 (0.1–2.4)	9 (3.3)	2.2 (0.6–7.4)	6 (2.2)	2.3 (0.6–9.1)	6.6 (0.9–46.7)	10.1 (1.0–99.1)*
Sleep	5 (2.4)	4 (1.5)	1.8 (0.4–8.0)	6 (2.2)	1.3 (0.3–5.9)	4 (1.5)	1.4 (0.3–6.8)	1.0 (0.2–4.6)	1.2 (0.2–7.8)
Number of equivalent years^a^ unloading containers	reference (*n* = 206)	0.0003–0.1 eq. years (*n* = 94)		0.11–0.9 eq. years (*n* = 89)		0.91–41.3 eq. years (*n* = 91)		0.11–0.9 eq. years (*n* = 89)	0.91–41.3 eq. years (*n* = 91)
Number of symptoms									
≥ 3 symptoms	78 (37.9)	22 (8.0)	0.5 (0.3–0.9)*	36 (13.1)	0.9 (0.5–1.5)	45 (16.4)	1.2 (0.7–2.2)	2.0 (1.0–3.9)	2.6 (1.3–5.1)**
≥ 5 symptoms	45 (21.8)	13 (4.7)	0.5 (0.2–1.0)	22 (8.0)	0.9 (0.5–1.8)	34 (12.4)	2.0 (1.0–3.8)*	2.1 (0.9–5.0)	4.0 (1.7–9.3)**
≥ 10 symptoms	12 (5.8)	3 (1.1)	0.5 (0.1–2.1)	5 (1.8)	1.0 (0.3–3.7)	14 (5.1)	4.0 (1.4–11.7)*	1.8 (0.3–9.5)	7.5 (1.7–32.8)**
Symptom domains (≥ 3)									
Neurological symptoms	9 (4.4)	1 (0.4)	0.2 (0.0–2.0)	7 (2.6)	1.8 (0.6–5.5)	5 (1.8)	1.3 (0.4–4.4)	2.4 (0.5–11.7)	2.1 (0.4–9.9)
Psychosomatic symptoms	20 (9.9)	5 (1.8)	0.4 (0.1–1.2)	9 (3.3)	0.7 (0.3–1.8)	15 (5.5)	1.1 (0.5–2.6)	1.5 (0.4–5.0)	3.0 (1.0–9.0)*
Mood	11 (5.3)	4 (1.5)	0.4 (0.1–1.5)	8 (2.9)	1.6 (0.5–4.5)	9 (3.3)	1.4 (0.5–4.1)	2.0 (0.5–8.0)	2.3 (0.6–8.6)
Memory/concentration	8 (3.9)	3 (1.1)	1.1 (0.2–5.3)	1 (0.4)	0.3 (0.0–2.9)	14 (5.1)	8.8 (2.5–31.4)***	0.7 (0.1–5.2)	6.8 (1.5–30.3)*
Fatigue	8 (3.9)	3 (1.1)	0.8 (0.2–3.6)	5 (1.8)	1.5 (0.4–5.5)	9 (3.3)	2.1 (0.6–7.3)	3.7 (0.6–21.0)	4.2 (0.8–21.3)
Sleep	5 (2.4)	4 (1.5)	1.8 (0.4–7.8)	5 (1.8)	1.6 (0.4–6.7)	5 (1.8)	1.3 (0.3–5.5)	1.2 (0.3–5.2)	1.1 (0.2–4.8)

All analyses were adjusted for age, ethnicity, smoking, alcohol consumption, education, personality traits and BMI

*p < 0.05, **p < 0.01, ***p < 0.001

^a^Equivalent years unloading is the time spend unloading containers over the work life expressed in years

When considering individual symptoms, we observed statistically significant associations with the exposure metric “equivalent years unloading containers” for symptoms in several domains (Table 4), but not for symptoms in the mood domain. A four-fold increased risk of the symptom ‘changes in sense of smell or taste’ (neurological symptoms domain) was shown for the highest tertile compared to the lowest exposure duration tertile. The risk of ‘sweating for no obvious reason’ was 2 and 3 times greater for the two highest tertiles when compared to the reference group and the lowest exposure tertile, respectively. The risk of ‘loss of sexual interest’ (psychosomatic symptoms domain) was 11 times greater for the highest exposure tertile compared to the reference group. Participants in the highest exposure tertile were four times more likely to report ‘forgetfulness’ (memory/concentration domain) and three times more likely to report ‘general weariness or tiredness’ (fatigue domain), when compared to the external reference group, respectively. Participants in the two highest tertiles were two times more likely to report’snoring that someone else has complained about’ (sleep domain) when compared to the group with the shortest duration (Table 4). However, further adjustment for alcohol consumption and BMI, which are contributing factors to excessive snoring, resulted in the association with snoring no longer being statistically significant (medium duration: OR 0.9, 95% CI 0.4–1.8; longest duration: OR 1.5, 95% CI 0.7–3.3).

**Table 4:** Prevalence odds ratios of individual neuropsychological symptoms for container handlers (n = 274) stratified by exposure duration (using number of equivalent years^1^ unloading containers)

Number of equivalent years^1^ unloading containers	External comparison (compared to the reference group)							Internal comparison (within container handlers)	
	Reference (*n* = 206)	0.0003–0.1 years (*n* = 94)		0.11–0.9 years (*n* = 89)		0.91–41.3 years (*n* = 91)		0.11–0.9 years (*n* = 89)	0.91–41.3 years (*n* = 91)
	*n* (%)	*n* (%)	OR (95% CI)	*n* (%)	OR (95% CI)	*n* (%)	OR (95% CI)	OR (95% CI)	OR (95% CI)
Domain and symptom									
Neurological symptoms									
Dropping things unintentionally	2 (1)	2 (2.1)	–	0 (0)	3.6 (0.3–41.4)	0 (0)	–	–	–
Weakness of your arms and feet	4 (1.9)	2 (2.1)	–	2 (2.3)	2.0 (0.4–10.0)	3 (3.3)	1.4 (0.3–6.6)	1.8 (0.3–10.0)	–
Decreased sensation in arms and legs	10 (4.9)	5 (5.3)	1.0 (0.3–3.3)	2 (2.3)	0.6 (0.2–2.3)	8 (8.9)	1.5 (0.5–3.9)	0.6 (0.1–2.8)	1.5 (0.4–5.2)
Numbness or heaviness in your arms or legs	8 (3.9)	1 (1.1)	–	3 (3.4)	0.8 (0.2–3.3)	3 (3.3)	1.0 (0.3–3.6)	0.8 (0.2–4.0)	–
Tingling in your arms or legs	6 (2.9)	4 (4.3)	0.4 (0.1–3.8)	2 (2.3)	1.3 (0.4–4.8)	5 (5.5)	1.6 (0.5–5.5)	3.0 (0.3–28.0)	3.6 (0.4–31.6)
Problems with balance	13 (6.3)	8 (8.5)	1.3 (0.5–3.6)	9 (10.2)	1.5 (0.6–3.7)	9 (9.9)	1.4 (0.6–3.4)	1.1 (0.4–3.2)	1.0 (0.4–2.9)
Changes in sense of smell or taste	17 (8.3)	4 (4.3)	0.5 (0.1–1.7)	13 (14.6)	1.6 (0.7–3.4)	14 (15.6)	1.9 (0.9–4.1)	3.5 (0.9–12.8)	4.2 (1.2–15.5)*
Decreased sensation on your face	3 (1.5)	2 (2.1)	0.8 (0.1–8.3)	2 (2.3)	2.0 (0.4–10.2)	2 (2.2)	1.2 (0.2–7.2)	2.1 (0.2–21.1)	1.4 (0.1–15.5)
Difficulties controlling your hand movements	13 (6.3)	3 (3.2)	0.3 (0.1–1.6)	6 (6.7)	1.1 (0.4–2.9)	3 (3.3)	0.4 (0.1–1.4)	3.4 (0.6–18.5)	1.1 (0.2–7.0)
Slowness in carrying out your daily activities	3 (1.5)	0 (0)	–	2 (2.3)	2.2 (0.3–16.2)	1 (1.1)	0.7 (0.1–7.2)	4.1 (0.3–54.2)	–
Trembling of hands	6 (2.9)	3 (3.2)	1.4 (0.3–5.6)	4 (4.5)	1.0 (0.2–4.0)	5 (5.5)	2.0 (0.6–6.4)	0.8 (0.1–3.9)	1.4 (0.3–5.9)
Psychosomatic symptoms									
Headache	11 (5.3)	5 (5.4)	0.9 (0.3–3.0)	7 (7.9)	1.3 (0.5–3.6)	10 (11)	1.7 (0.7–4.3)	1.4 (0.4–5.1)	2.0 (0.6–6.7)
Sweating for no obvious reason	15 (7.3)	6 (6.5)	0.7 (0.2–2.2)	12 (13.5)	1.7 (0.8–3.8)	13 (14.3)	2.2 (1.0–4.9)*	2.5 (0.7–8.3)	3.2 (1.0–10.4)*
Nausea e.g., feeling sick in your stomach	18 (8.7)	7 (7.5)	0.7 (0.3–2.1)	12 (13.5)	1.7 (0.8–3.5)	9 (9.9)	1.0 (0.4–2.4)	2.2 (0.7–6.6)	1.4 (0.4–4.3)
Stomach pains	17 (8.3)	6 (6.4)	0.4 (0.1–1.6)	9 (10.1)	1.3 (0.6–3.0)	5 (5.5)	0.7 (0.2–1.8)	2.8 (0.7–10.6)	1.5 (0.4–6.2)
Dizziness	2 (1)	1 (1.1)	1.2 (0.1–16.3)	2 (2.3)	4.5 (0.4–48.7)	2 (2.2)	2.7 (0.3–26.2)	2.5 (0.2–33.5)	1.7 (0.1–20.7)
Shortness of breath without physical exertion	19 (9.3)	7 (7.5)	0.8 (0.3–2.0)	9 (10.1)	1.1 (0.5–2.4)	11 (12.1)	1.0 (0.5–2.3)	1.5 (0.5–4.6)	1.4 (0.5–4.0)
Heart fluttering (palpitations)	18 (8.8)	6 (6.4)	0.4 (0.1–1.6)	10 (11.4)	1.5 (0.7–3.3)	8 (8.8)	1.0 (0.4–2.4)	3.2 (0.8–11.8)	2.5 (0.6–9.6)
Ringing in your ears (tinnitus)	19 (9.2)	5 (5.3)	0.5 (0.2–1.5)	6 (6.7)	0.6 (0.2–1.6)	8 (8.8)	0.8 (0.3–1.9)	1.2 (0.3–4.6)	1.6 (0.5–5.5)
Feeling of general exhaustion	9 (4.4)	2 (2.1)	0.5 (0.1–2.3)	3 (3.4)	0.7 (0.2–2.7)	8 (8.8)	1.6 (0.6–4.5)	2.1 (0.3–15.8)	4.3 (0.8–24.6)
Loss of sexual interest	1 (0.5)	2 (2.2)	4.7 (0.4–54.1)	2 (2.3)	4.6 (0.4–54.2)	6 (6.6)	10.8 (1.2–94.3)*	0.8 (0.1–6.4)	2.1 (0.4–11.1)
Lowered alcohol tolerance	18 (9.1)	4 (4.4)	0.3 (0.1–1.2)	3 (3.5)	0.5 (0.2–1.5)	10 (11.1)	1.1 (0.5–2.4)	2.1 (0.4–11.8)	4.1 (0.8–19.5)
Diarrhoea	1 (0.5)	0 (0)	–	0 (0)	–	1 (1.1)	1.7 (0.1–31.7)	–	–
Constipation	5 (2.4)	6 (6.4)	2.0 (0.5–7.7)	5 (5.6)	2.8 (0.8–9.6)	3 (3.3)	1.4 (0.4–5.4)	1.4 (0.4–5.4)	0.7 (0.2–3.0)
Loss of appetite	6 (2.9)	1 (1.1)	0.5 (0.1–3.9)	2 (2.3)	0.7 (0.1–3.3)	3 (3.3)	1.0 (0.2–4.4)	1.4 (0.1–15.7)	2.3 (0.2–22.9)
Feeling of a tight band around your head	5 (2.4)	1 (1.1)	0.5 (0.1–4.5)	4 (4.5)	1.6 (0.4–6.2)	6 (6.6)	2.3 (0.7–7.9)	3.4 (0.4–32.5)	4.6 (0.5–39.1)
Mood									
Difficulty getting started at work	8 (3.9)	1 (1.1)	0.3 (0.0–2.7)	0 (0)	–	2 (2.2)	0.5 (0.1–2.6)	–	1.9 (0.1–25.6)
Feeling irritable	12 (5.8)	4 (4.3)	0.3 (0.1–1.7)	6 (6.7)	1.3 (0.5–3.5)	5 (5.5)	0.7 (0.2–2.0)	4.2 (0.8–23.6)	2.1 (0.4–11.6)
Feeling depressed	3 (1.5)	2 (2.1)	0.7 (0.1–7.9)	3 (3.4)	2.7 (0.5–15.7)	1 (1.1)	1.1 (0.2–7.6)	4.5 (0.4–55.9)	1.7 (0.1–21.7)
Feeling impatient	20 (9.7)	7 (7.5)	0.5 (0.2–1.5)	4 (4.5)	0.5 (0.2–1.3)	12 (13.2)	1.2 (0.6–2.7)	1.0 (0.2–3.8)	2.6 (0.8–8.6)
Being upset by trivial things	10 (4.9)	6 (6.4)	0.9 (0.3–3.2)	2 (2.3)	0.6 (0.2–2.4)	6 (6.6)	1.0 (0.4–3.0)	0.7 (0.1–3.3)	1.1 (0.3–4.4)
Feeling restless	11 (5.4)	1 (1.1)	0.2 (0.0–1.9)	2 (2.3)	0.3 (0.1–1.6)	5 (5.5)	0.9 (0.3–2.6)	2.1 (0.2–26.5)	4.1 (0.4–38.0)
Rapid changes in mood	7 (3.4)	4 (4.3)	0.7 (0.1–3.4)	3 (3.4)	0.9 (0.2–3.6)	3 (3.3)	1.0 (0.3–3.6)	1.4 (0.2–9.1)	1.5 (0.3–8.9)
Feeling of detachment	18 (8.7)	11 (11.8)	1.5 (0.6–3.5)	11 (12.5)	1.3 (0.6–2.9)	7 (7.7)	0.8 (0.3–1.9)	0.9 (0.3–2.3)	0.5 (0.2–1.4)
Lack of drive, energy, enthusiasm	7 (3.4)	2 (2.1)	0.3 (0.0–2.7)	3 (3.4)	0.9 (0.2–3.7)	4 (4.4)	1.3 (0.4–4.3)	4.2 (0.4–49.5)	4.9 (0.5–48.1)
Lack of interest in social activities	14 (6.8)	5 (5.3)	0.6 (0.2–2.0)	7 (8)	1.2 (0.5–3.1)	6 (6.7)	0.7 (0.3–1.9)	1.8 (0.5–6.4)	1.1 (0.3–4.2)
Difficulty in controlling anger	5 (2.4)	4 (4.3)	2.3 (0.6–9.2)	1 (1.1)	0.4 (0.0–3.3)	3 (3.3)	1.2 (0.3–5.4)	0.2 (0.0–1.5)	0.5 (0.1–2.5)
Memory/concentration									
Forgetfulness	6 (2.9)	2 (2.1)	0.8 (0.1–4.1)	3 (3.4)	1.1 (0.3–4.5)	11 (12.1)	3.8 (1.3–11.0)*	1.4 (0.2–8.9)	4.8 (1.0–23.3)
Having to write notes to remember things	18 (8.8)	6 (6.4)	0.9 (0.3–2.3)	7 (7.9)	0.6 (0.2–1.7)	9 (9.9)	1.0 (0.5–2.4)	0.7 (0.2–2.4)	1.2 (0.4–3.6)
Forgetting what you were about to say or do	7 (3.4)	1 (1.1)	0.3 (0.0–2.7)	1 (1.1)	0.3 (0.0–2.5)	8 (8.8)	2.1 (0.7–6.2)	1.0 (0.1–16.5)	6.7 (0.8–57.3)
Difficulty in concentrating	4 (2)	2 (2.2)	1.3 (0.2–7.6)	3 (3.4)	1.1 (0.2–6.4)	4 (4.4)	2.3 (0.5–9.5)	0.8 (0.1–6.7)	1.8 (0.3–10.3)
Daydreaming	11 (5.3)	6 (6.4)	1.1 (0.4–3.5)	10 (11.2)	2.3 (0.9–5.6)	10 (11)	1.7 (0.7–4.4)	2.3 (0.7–7.6)	1.6 (0.5–5.1)
Feeling confused when try to concentrate	2 (1)	2 (2.1)	2.5 (0.3–18.6)	0 (0)		3 (3.3)	2.7 (0.4–17.1)	–	1.1 (0.2–7.0)
Difficulty remembering names and dates	14 (6.8)	9 (9.6)	1.5 (0.6–3.8)	6 (6.7)	0.7 (0.2–2.0)	10 (11)	1.6 (0.7–3.6)	0.4 (0.1–1.5)	1.1 (0.4–2.8)
Absent-mindedness	2 (1)	2 (2.1)	2.5 (0.3–18.3)	2 (2.3)	1.1 (0.1–12.4)	3 (3.3)	3.7 (0.7–21.3)	0.4 (0.0–5.4)	1.5 (0.2–9.1)
Difficulty remembering what read/TV	5 (2.4)	0 (0)		4 (4.5)	1.2 (0.3–5.3)	6 (6.6)	2.8 (0.8–9.3)	0.4 (0.1–1.7)	
Other people complain about your memory	12 (5.8)	3 (3.2)	0.6 (0.2–2.3)	3 (3.4)	0.5 (0.1–1.8)	8 (8.8)	1.3 (0.5–3.3)	0.8 (0.1–4.0)	2.1 (0.5–8.2)
Fatigue									
Falling asleep when not in bed	6 (2.9)	2 (2.1)	0.9 (0.2–4.5)	0 (0)	–	2 (2.2)	0.6 (0.1–3.0)	–	0.7 (0.1–5.0)
Unusual tiredness in the evening	17 (8.3)	5 (5.3)	0.5 (0.1–1.6)	7 (7.9)	0.4 (0.1–1.4)	7 (7.7)	1.1 (0.5–2.6)	1.0 (0.2–4.3)	2.3 (0.7–8.0)
Sleepiness	17 (8.3)	5 (5.4)	0.5 (0.1–1.6)	4 (4.5)	0.3 (0.1–1.2)	9 (9.9)	1.1 (0.5–2.6)	0.8 (0.1–4.0)	2.5 (0.7–9.5)
Feeling tired when you wake up	12 (5.9)	6 (6.4)	1.3 (0.5–3.8)	8 (9)	1.2 (0.4–3.2)	9 (9.9)	1.7 (0.7–4.4)	1.0 (0.3–3.2)	1.3 (0.4–3.9)
Lack of energy	8 (3.9)	2 (2.1)	0.5 (0.1–2.5)	1 (1.1)	0.3 (0.0–2.4)	7 (7.8)	1.7 (0.5–5.4)	0.9 (0.1–13.7)	5.8 (0.8–44.7)
General weariness (or tiredness)	9 (4.4)	5 (5.4)	0.9 (0.3–3.6)	5 (5.6)	1.4 (0.4–4.6)	12 (13.2)	3.0 (1.1–8.1)*	1.6 (0.4–7.5)	3.5 (0.9–13.3)
Needing more sleep than you used to	6 (2.9)	3 (3.2)	1.2 (0.3–5.0)	2 (2.3)	0.8 (0.1–4.0)	3 (3.3)	0.9 (0.2–3.6)	0.8 (0.1–5.4)	0.8 (0.1–4.2)
Sleep									
Difficulty falling asleep	11 (5.3)	7 (7.5)	1.5 (0.5–4.3)	7 (7.9)	1.3 (0.5–3.5)	10 (11)	2.1 (0.9–5.3)	1.0 (0.3–3.1)	1.4 (0.5–4.3)
Broken sleep	15 (7.3)	11 (11.7)	1.8 (0.7–4.3)	8 (9)	0.9 (0.4–2.4)	11 (12.1)	1.6 (0.7–3.6)	0.6 (0.2–1.6)	0.9 (0.4–2.3)
Waking up too early	12 (5.8)	7 (7.5)	1.5 (0.6–4.0)	7 (7.9)	0.8 (0.3–2.5)	7 (7.7)	1.3 (0.5–3.3)	0.6 (0.2–2.1)	0.9 (0.3–2.7)
Nightmares	16 (7.8)	9 (9.6)	1.3 (0.5–3.2)	4 (4.5)	0.6 (0.2–1.7)	8 (8.8)	0.9 (0.3–2.1)	0.4 (0.1–1.5)	0.7 (0.2–2.0)
Snoring someone else has complained about	22 (10.7)	11 (11.7)	0.8 (0.3–2.0)	15 (16.9)	1.4 (0.7–2.9)	17 (18.7)	2.0 (1.0–3.9)*	1.8 (0.7–4.8)	2.6 (1.0–6.6)*

All analyses were adjusted for age and personality traits

*p < 0.05, **p < 0.01, ***p < 0.001

For acute symptoms, no statistically significant differences between the reference and the exposed group were observed (Table 5). For self-reported sensitivity to environmental conditions (Table 5), exposed workers and all sub-groups, except the log workers, were less likely to report to be sensitive to heat compared to the reference group. The overall group of exposed workers, and the subgroup of container handlers were also less likely to report to be sensitive to bright lights compared to the reference group.

**Table 5:** Prevalence odds ratios of acute symptoms and sensitivity to environmental conditions comparing exposed with reference workers

	Reference group (*n* = 206)	Exposed group (*n* = 365)		Each occupation within the exposed group (compared to the reference group)							
				Container handler (*n* = 274)		Retail worker (*n* = 38)		Log worker (*n* = 18)		Fumigator (*n* = 35)	
	*n* (%)	*n* (%)	OR (95% CI)	*n* (%)	OR (95% CI)	*n* (%)	OR (95% CI)	*n* (%)	OR (95% CI)	*n* (%)	OR (95% CI)
Acute symptoms											
Irritation of the eyes	17 (8.3)	31 (8.5)	1.2 (0.6–2.4)	24 (8.8)	1.3 (0.6–2.7)	1 (2.6)	0.4 (0.0–3.2)	4 (22.2)	3.5 (0.9–13.3)	2 (5.7)	0.5 (0.1–4.0)
Feeling drunk without drinking alcohol	0	2 (0.5)	–	2 (0.7)	–	0	–	0	–	0	
Dryness of the mouth or throat	22 (10.7)	23 (6.3)	0.6 (0.3–1.2)	19 (6.9)	0.7 (0.3–1.4)	2 (5.3)	0.5 (0.1–2.3)	1 (5.6)	0.6 (0.1–5.0)	1 (2.9)	
Throat irritation	9 (4.4)	16 (4.4)	1.7 (0.6–4.8)	13 (4.7)	1.9 (0.6–5.5)	1 (2.6)	0.8 (0.1–7.6)	1 (5.6)	3.3 (0.3–33.9)	1 (2.9)	1.5 (0.2–13.6)
Runny nose	20 (9.8)	19 (5.2)	0.5 (0.3–1.1)	15 (5.5)	0.6 (0.3–1.2)	1 (2.6)	0.2 (0.0–1.9)	2 (11.1)	1.5 (0.3–7.9)	1 (2.9)	0.4 (0.0–3.1)
Unpleasant taste in mouth	5 (2.4)	11 (3.0)	1.3 (0.4–4.8)	9 (3.3)	1.6 (0.4–6.1)	0	–	1 (5.6)	4.4 (0.4–50.8)	1 (2.9)	
Sensitivity to environmental conditions											
Bright lights	70 (34)	96 (26)	0.6 (0.4–0.9)*	71 (26)	0.6 (0.4–0.9)*	12 (32)	0.8 (0.3–1.8)	6 (33.3)	0.9 (0.3–2.6)	7 (20)	0.4 (0.2–1.1)
Loud music or other loud noises	49 (24)	81 (22)	0.8 (0.5–1.3)	62 (23)	0.8 (0.5–1.3)	7 (18)	0.5 (0.2–1.6)	5 (27.8)	1.1 (0.3–3.6)	7 (20)	0.8 (0.3–2.2)
Strong smells	79 (39)	132 (36)	0.8 (0.5–1.2)	106 (39)	0.9 (0.6–1.3)	8 (21)	0.3 (0.1–0.8)*	8 (44.4)	1.1 (0.4–3.1)	10 (28.6)	0.6 (0.3–1.4)
Rough fabrics next to my skin	62 (30)	111 (31)	0.8 (0.5–1.2)	87 (32)	0.9 (0.6–1.3)	8 (21)	0.5 (0.2–1.3)	6 (33.3)	0.6 (0.2–1.9)	10 (28.6)	1.0 (0.4–2.2)
Heat	81 (40)	116 (32)	0.5 (0.4–0.8)**	95 (35)	0.6 (0.4–1.0)*	8 (21)	0.3 (0.1–0.7)**	5 (27.8)	0.5 (0.2–1.4)	8 (22.9)	0.4 (0.2–1.0)*
Cold	73 (36)	123 (34)	0.9 (0.6–1.4)	90 (33)	0.9 (0.6–1.3)	14 (37)	0.9 (0.4–1.9)	7 (38.9)	1.1 (0.4–3.1)	12 (34.3)	1.2 (0.5–2.6)
Tobacco smoke	79 (39)	145 (40)	1.0 (0.7–1.6)	111 (41)	1.0 (0.7–1.6)	15 (40)	0.9 (0.4–2.1)	6 (33.3)	1.2 (0.4–4.1)	13 (37.1)	1.1 (0.5–2.6)
Certain foods	42 (21)	78 (21)	0.9 (0.5–1.4)	61 (22)	0.9 (0.5–1.5)	6 (16)	0.5 (0.2–1.4)	5 (27.8)	1.4 (0.4–4.3)	6 (17.1)	1.0 (0.4–2.6)

All analyses were adjusted for age, ethnicity, smoking, alcohol consumption, education, personality traits and BMI

*p < 0.05, **p < 0.01, ***p < 0.001

Several workers in the exposed group reported night shift work (*n* = 77), whilst none in the reference group reported this. As this may have an effect on neuropsychological symptoms, we repeated the analyses related to the results described in Tables 2 and 3 excluding all night shift workers. This did not appreciably alter the results (data not shown).

## Discussion

This is one of only a few health surveys assessing (non-acute) neuropsychological symptoms in workers exposed to fumigants and chemicals off-gassed from cargo of shipping containers. Container handlers overall did not report more symptoms than the reference group, but those handling containers for more than 10 years were more likely to report symptoms, both when compared to an external reference group and when compared to container handlers with shorter employment in the industry. The duration of container handling was particularly associated with symptoms in the memory/concentration domain. Retail workers were more likely to report ≥ 10 symptoms overall, and they were also more likely to report fatigue symptoms.

Consistent with our study, one of two other studies on non-acute health effects in container handlers (Lucas et al. 2019) found a relatively low prevalence of symptoms, which the authors attributed to the healthy worker effect. Our results provide further evidence for this, as workers in the shortest duration group were less likely to report symptoms compared to the reference group (Tables 3 and 4), suggesting that workers entering the highly physical occupation of container handler may indeed be healthier.

The previous study, which was relatively small and did not include a reference group, assessed duration-response associations, or controlled for potential confounders, also found that workers exposed to fumigants in the past years were more likely to report memory disorders (Lucas et al. 2019). This is consistent with the positive duration–response association we observed for symptoms in the memory/concentration domain. This finding is also consistent with a study on methyl bromide and sulfuryl fluoride exposed structural fumigators (fumigation of structures such as houses) who were shown to perform worse on the ‘Pattern Memory’ test compared to a reference group (Calvert et al. 1998); the same study also found an association between methyl bromide exposure and poorer test results for the ‘logical memory test of the Wechsler Memory Scale’. In addition, memory/concentration symptoms have also frequently been reported as a late and sometimes chronic symptom in case reports of acute intoxications (Burgess et al. 2000; de Souza et al. 2013; Verschoor et al. 2010, 2011). Taken together, these findings suggest that workers exposed to fumigants may be particularly at risk of experiencing symptoms in the memory/concentration domain. However, a recent study of neuropsychological symptoms in 165 logistic transport workers who were involved in transportation and storage of goods, but not necessarily entered containers, did not confirm this. It found that, compared to an external reference group, workers were significantly less likely to report ‘forgetfulness’; they were also less likely to report ‘difficulty in concentrating’, although this did not reach statistical significance (Lovas et al. 2021). However, the exposed and reference group in this study differed strongly in gender distribution (92.6% males in the exposed group compared to 20.6% in the unexposed group), and although this was adjusted for, some residual confounding cannot be excluded. Therefore, although the evidence is mixed, if exposure to fumigants is indeed associated with symptoms in the memory/concentration domain as suggested in our study, then this is important in itself, but it may also contribute to additional risks for workers. In particular, deficits in memory and concentration may contribute to an increased risk of work-related injuries, with cargo handling ranked as one of the highest potential accident risk activities in container ports (Sunaryo and Hamka 2017). It also has the potential to increase the risk of accidents while driving home from work.

Container handlers with longer exposure duration were more likely to report ‘sweating for no obvious reasons’ (Table 4). Body temperature and sweating are regulated by the autonomic nervous system (McCorry 2007) and previous research in fumigators comparing pre- and post-work heart rate variability (HRV), a measure to assess autonomic dysfunction, has shown a significant association between methyl bromide exposure and reduced HRV indices (Choi et al. 2021). Although speculative, this suggests that our finding may be explained by fumigant (or other chemical) exposure.

Container handlers with longer exposure duration were also more likely to report ‘changes in sense of smell or taste’ (Table 4). Olfactory dysfunction has previously been attributed to exposure to formaldehyde and possibly styrene, as well as several fumigants (methyl bromide, sulfuryl fluoride and chloropicrin) (Calvert et al. 1998; Werner and Nies 2018). However, as these fumigants are often used together, it is difficult to differentiate between the effects of these chemicals (Calvert et al. 1998; Werner and Nies 2018). Exposure duration was also associated with ‘loss of sexual interest’ (Table 4). While this may not be directly related, erectile dysfunction has also been reported in case reports on fumigators (Magnavita 2009; Park et al. 2005).

The positive associations observed between exposure duration and neuropsychological symptoms in container handlers suggest that long-term exposure may be important. We have previously reported personal full-shift exposure measurements for 133 container handlers who were also included in the current study, with levels generally below current occupational exposure limits for most measured fumigants and residual chemicals, except formaldehyde (Hinz et al. 2020) (Table 6). This suggests that symptoms may occur after long-term exposure at levels below current occupational standards, possibly due to the combined effect of multiple chemical exposures, or repeated incidental high peak exposures. Alternatively, symptoms may be attributable to other chemicals not measured as part of the panel tested. It is also possible that symptoms may be due to high historical exposures.

**Table 6:** Previously reported personal 8-h exposures for container handlers (*n* = 133) using 2020 occupational exposure limits (WES and TLV) (adapted from Hinz et al. 2020)

Chemical (ppb^a^)	WES^b^	TLV^c^	LoD^d^	< LoD (%)	> WES	> TLV
Fumigants						
1,2-Dibromoethane	n/a^e^	n/a	5	71.4	n/a	n/a
Chloropicrin	100	100	5	88.7	0	0
Ethylene oxide	100	1000	10	88.7	1 (0.8%)	0
Hydrogen cyanide	10000^f^	4700^f^	3	78.9	0	0
Hydrogen phosphide	300	50	3	75.9	0	0
Methyl bromide	5000	1000	5	66.2	0	0
Non-fumigants						
1,2-Dichloroethane	5000	10,000	5	79.7	0	0
C2-alkylbenzenes	50,000	20,000	5	51.9	0	0
Acetaldehyde	20000^f^	25,000 ^f^	25	61.7	0	0
Ammonia	25,000	25,000	15	83.5	0	0
Benzene	50	500	5	88.0	0	0
Formaldehyde^g^	300	100	25	44.7	4 (3.3%)	36 (29.3%)
Styrene	20,000	10,000	2	92.5	0	0
Toluene	50,000	20,000	3	29.3	0	0

^a^*ppb* parts per billion

^b^8-h Workplace Exposure Standards (WES) set by WorkSafe NZ (2019)

^c^8-h Workplace Exposure Standards (TLV-Threshold LIMIT Value) set by American Conference of Governmental Industrial Hygienists (2020)

^d^Limit of detection

^e^n/a: not applicable because the WES is below the LoD and the TLV is not set by the American Conference of Governmental Industrial Hygienists

^f^These chemicals do not have an 8-h Workplace Exposure Standard but only a ceiling limit which was used instead

^g^Formaldehyde analyses were available for only 123 samples

The study included an external reference group of construction workers, which was comparable to the exposed workers in terms of age, smoking, working hours, and occupational physical activity. Nonetheless, the groups differed in terms of alcohol consumption, and some minor differences in education and BMI were also observed. However, analyses were adjusted for these factors, which are therefore unlikely to explain the associations observed. Importantly, similar associations were observed in internal analyses that did not rely on the external reference group.

Only a small number of participants in occupations other than container handlers were included, limiting the ability to detect associations for these workers. Nonetheless, we did observe an increased risk of reporting ≥ 10 symptoms and for reporting fatigue symptoms for the group of retail workers; however, associations with exposure duration could not be determined. Thus, results remain largely inconclusive for retail workers. Nonetheless, given the potential exposures to fumigants and other hazardous substances, and possible health risks that these workers may experience, we consider that further research focusing on this common occupational group is warranted.

Other limitations include the cross-sectional nature of the study and the fact that neurobehavioral symptoms were self-reported and not objectively assessed. Also, due to low number of females in the exposed group, except in the subgroup of retail workers, and only two females in the reference group, we excluded all female participants. It therefore remains unclear whether results can be extrapolated to female workers. Also, although one-off exposure measurements were available for a proportion of the study populations (see above), this was insufficiently detailed; this study can therefore not indicate which specific exposures may be responsible for the observed associations. Finally, analyses of individual symptoms were adjusted for age and personality trait score only (due to non-convergence of fully adjusted models), so for those analyses confounding by other factors cannot be excluded.

In conclusion, this study suggests that container handlers may have an increased risk of neuropsychological symptoms, particularly related to memory and concentration. Retail workers may also be at risk of neuropsychological symptoms, but due to the relatively small population size, results are inconclusive.

## Data Availability

The datasets generated during and analysed during the current study are available from the corresponding author on reasonable request.
